# Influence of low gravity on the penetration resistance of lunar regolith

**DOI:** 10.1038/s41526-026-00562-8

**Published:** 2026-01-21

**Authors:** Jun Chen, Ruilin Li, Shigen Fu

**Affiliations:** 1 Institute of Mine Safety, China Academy of Safety Science and Technology, Beijing, China; 2 State Key Laboratory of Intelligent Construction and Healthy Operation and Maintenance of Deep Underground Engineering, China University of Mining and Technology, Xuzhou, China

**Keywords:** Engineering, Physics

## Abstract

Lunar surface operations conducted by the United States and the Soviet Union confirmed that penetration resistance is a key indicator for evaluating the engineering properties of lunar regolith. To quantitatively assess the influence of reduced gravity on penetration resistance, this study employed a newly developed Geotechnical Magnetic-gravity Modeling Test (GMMT) system to perform cone penetration tests under controlled gravitational acceleration levels of 1/6 g, 1 g, and 2 g. The results indicated that the normalized penetration resistance increased as gravity decreased, and this effect was amplified at higher relative densities. To investigate the underlying mechanisms, discrete element method (DEM) simulations were conducted. The findings revealed that, in addition to gravity, in situ factors such as high relative density and irregular particle morphology also significantly enhanced penetration resistance by strengthening interparticle contact and friction. These non-gravitational effects partially offset the expected reduction in resistance under lower gravity, leading to a smaller-than-anticipated decline. This study provides new insights into the gravity-dependent penetration behavior of lunar regolith.

## Introduction

Despite rapid advancements in space technology, knowledge of the geomechanical properties of lunar regolith remains limited. This limitation has contributed to operational challenges in previous missions: the Apollo 15 mission failed to reach the intended depth and encountered difficulty retrieving core stems^1^; the Luna 20 mission was halted at a depth of 25 cm^2^; and the Chang’e-5 mission experienced unexpectedly high resistance at around 1 m depth, resulting in a lower-than-expected sample mass^3^. As upcoming missions involve more complex tasks, including lunar base construction^4–6^ and in situ resource utilization^7–9^, a deeper understanding of regolith mechanical behavior is essential for mission success^10–14^. Cone Penetration Testing (CPT) is a widely used technique for evaluating subsurface mechanical properties^15,16^. Its effectiveness has been demonstrated during lunar exploration missions conducted by the United States and the former Soviet Union^17,18^. Accordingly, this method has garnered increasing attention^19–21^. However, most physical CPT tests have been conducted under Earth’s gravity. Given that Earth’s gravity is approximately six times that of the Moon, the applicability of these results to lunar conditions remains uncertain^22,23^. Although several studies have attempted to simulate penetration under different gravitational acceleration levels using the DEM method, such numerical simulations generally lack validation against corresponding physical experiments, leaving their reliability in question^24,25^. Additionally, some researchers have utilized parabolic flight campaigns to conduct penetration tests in reduced-gravity environments^26,27^. However, the extremely short duration of low-gravity conditions necessitates high-speed penetration tests, which deviate significantly from the slow penetration processes expected on the lunar surface, thereby limiting their practical relevance.

The 1/6 g gravity is a key factor influencing the penetration resistance of in situ lunar regolith. Recreating this gravity environment is essential for accurately capturing its penetration characteristics, and three physical methods can theoretically simulate the low-gravity conditions for cone penetration tests. Drop tower works by releasing partial gravitational acceleration to achieve the target gravity environment. Systems of this kind have been constructed in several countries, e.g., the United States^28,29^, Germany^30,31^, China^32,33^, Japan^34^, and India^35^. However, due to the height limitations of such towers, the 1/6 g condition is sustained for only a few seconds, which is insufficient for standard penetration testing. Reduced-gravity aircraft operates on a principle similar to that of a drop tower, typically sustaining 1/6 g for up to 30 s. Representative reduced-gravity aircraft include the Zero-G aircraft^36^, Ilyushin Il-76^37^, and Falcon 20^38^. Nevertheless, this duration remains insufficient for cone penetration testing, and the physiological demands placed on researchers further limit its widespread use. Magnetic levitation method (GMMT method) employs uniform and stable magnetic forces to counteract partial gravity, thereby creating a desired reduced-gravity environment. In earlier work, Professor Geim^39^ demonstrated the feasibility of this approach by levitating a frog using magnetic fields. This unconventional experiment was later recognized with the 2000 Ig Nobel Prize in Physics. In 2021, Sanavandi and Guo^40^ proposed a superconducting magnet coil structure, aiming to generate variable-gravity conditions over a larger spatial region. However, no physical realization of their specific design has been reported to date.

The limitations of current reduced-gravity simulation methods have resulted in most physical penetration tests being performed under Earth’s gravity, which has hindered in-depth exploration of penetration resistance behavior under low-gravity conditions. To address this gap, this study conducted static cone penetration tests under 1/6 g, 1 g, and 2 g conditions using a self-developed magnetic levitation system^41^ and the magnetic CUMT-1 lunar regolith simulant^42^. The results were then compared and analyzed with those from real lunar regolith and other simulants. Subsequently, the discrete element method was employed to quantitatively analyze the evolution of force chains during penetration, providing insights into the mesoscopic mechanisms through which gravity affects penetration resistance.

## Results

### Physical cone penetration test results under three gravitational acceleration levels

Physical cone penetration tests of the CUMT-1 lunar regolith simulant were conducted using the GMMT method at three gravitational acceleration levels (1/6 g, 1 g, and 2 g) and three relative densities (40%, 58%, and 76%). The penetration resistance *q* and normalized penetration resistance *Q* were utilized to quantify the penetration characteristics of the CUMT-1 lunar regolith simulant, with definitions provided as follows:

1 $$q=\frac{F}{A}$$

2 $$Q=\frac{q}{\rho gz}$$

3 $$Z=\frac{z}{B}$$

where *F* is the force exerted on the drill rod, *A* is the maximum cross-sectional area of the cone tip in the horizontal direction, ^*ρ*^ is the bulk density of CUMT-1 lunar regolith simulant under a specific relative density condition, *g* is the gravitational acceleration under a specific gravitational field, *z* is the penetration depth of the cone tip, *B* is the edge length of the cone tip, and *Z* is the normalized penetration depth.

**Fig. 1:**
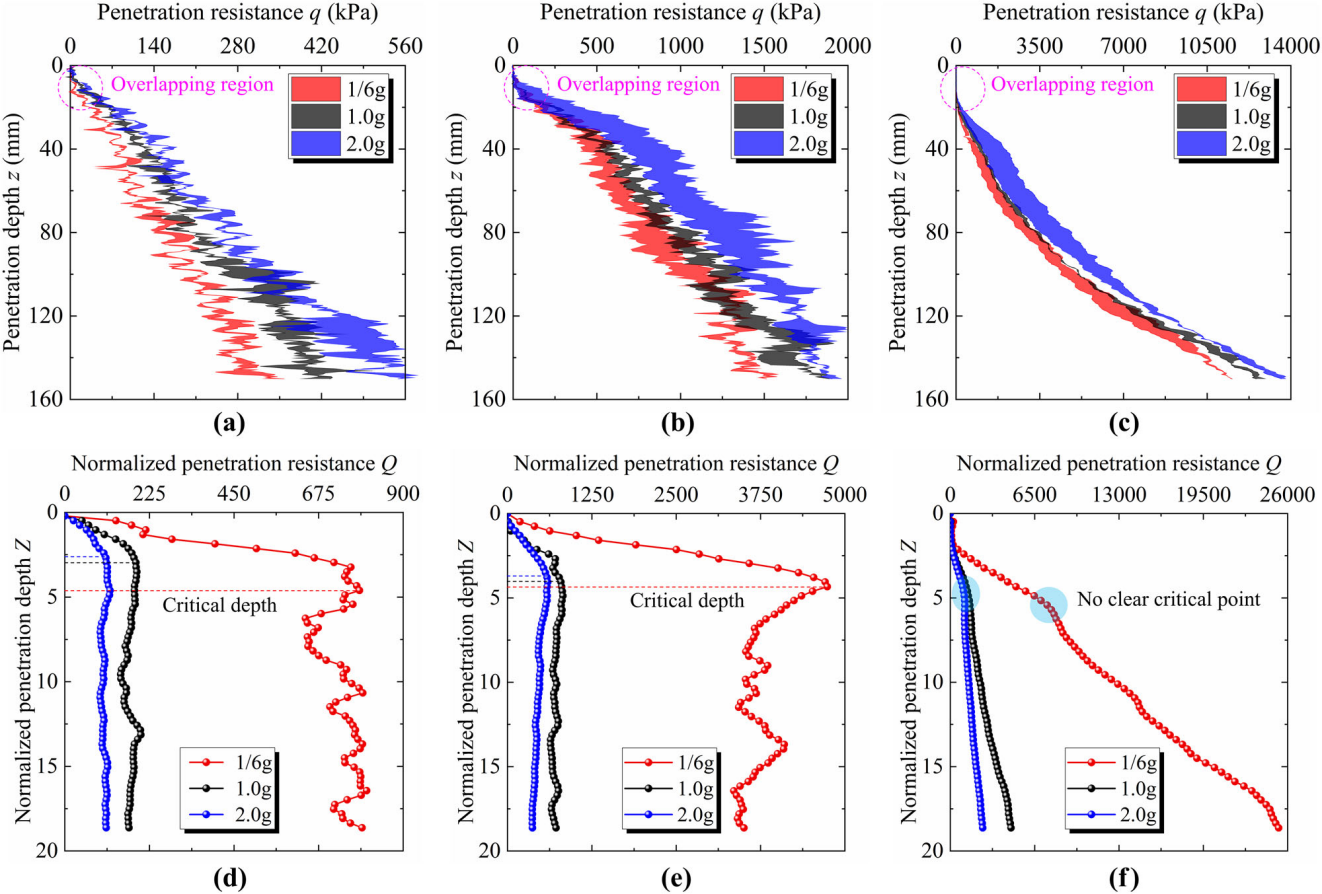
Penetration resistance and normalized penetration resistance curves of CUMT-1 lunar regolith simulant under three gravities and three relative densities (physical tests). **a** Penetration resistance curves at the relative density of 40%. **b** Penetration resistance curves at the relative density of 58%. **c** Penetration resistance curves at the relative density of 76%. **d** Normalized penetration resistance curves at the relative density of 40%. **e** Normalized penetration resistance curves at the relative density of 58%. **f** Normalized penetration resistance curves at the relative density of 76%.

Penetration tests were conducted under three gravitational acceleration levels and three relative density conditions. At least two parallel trials were performed under each condition to ensure the reproducibility of the results. When a noticeable discrepancy was observed between the two tests, a third trial was carried out to further verify data reliability. Figure 1a–c presents the results obtained after filling the area between the two parallel penetration curves. It can be seen that certain fluctuations exist between the parallel tests under the same conditions, which mainly result from nonuniformity in the soil fabric during penetration. Despite these fluctuations, the observed variability does not affect the analysis of the gravity effect. The penetration resistance increases steadily with increasing gravity under all three relative density conditions. Furthermore, across all relative density conditions, the penetration curves corresponding to different gravitational acceleration levels overlap during the initial stage. This phenomenon likely arises from the relatively low gravity-induced vertical stress within a depth of 40 mm, exerting only a limited influence on the overall resistance. At this stage, penetration resistance is primarily governed by relative density and exhibits some variability due to limitations in the sample preparation process.

Furthermore, under the identical relative density conditions, penetration resistance decreases with decreasing gravitational acceleration level, aligning well with established expectations. However, as relative density increases, the influence of gravity on penetration resistance becomes progressively less pronounced. For example, at a relative density of 40%, the average penetration resistance within a depth of 150 mm under 1/6 g gravity is ~29.1% lower than that under 1 g gravity. In contrast, the reduction is only about 8.8% at a relative density of 76%. According to previous lunar exploration data^43^, the in situ relative density of lunar regolith increases rapidly with depth, reaching up to about 92% at ~60 cm. This suggests that the reduction in resistance caused by the 1/6 g lunar gravity may be substantially less than previously expected.

The normalized penetration resistance curves are presented in Fig. 1d-e. It can be observed that the normalized penetration resistance increases at an accelerating rate as the gravitational acceleration decreases and the relative density increases. Specifically, when the relative densities are 40%, 58%, and 76%, the average increase in normalized penetration resistance within a depth of 150 mm caused by a reduction in gravity from 1 g to 1/6 g is approximately 308.2%, 429.1%, and 439.2%, respectively. Meanwhile, at fixed gravitational acceleration levels of 1/6 g, 1 g, and 2 g, increasing the relative density from 40% to 76% results in average increases in normalized penetration resistance of approximately 1686.1%, 1252.6%, and 1242.4%, respectively. These results indicate that the normalized penetration resistance is highly sensitive to both gravitational acceleration and relative density. However, based on the magnitude of the increases, it is evident that when the normalized penetration depth is less than 20, relative density exerts a considerably greater influence on normalized penetration resistance than gravity.

### DEM simulation results of cone penetration under gravitational acceleration levels

**Fig. 2:**
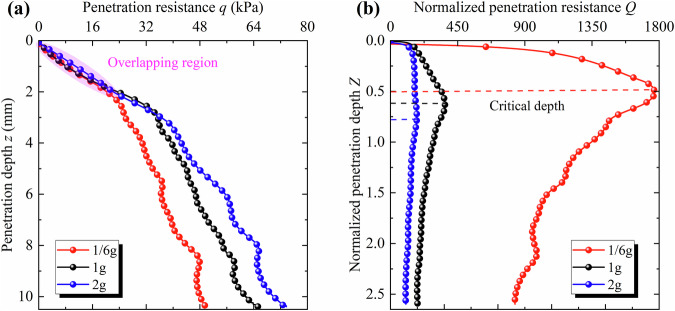
Penetration resistance and normalized penetration resistance curves under three gravities and two relative densities (numerical tests). **a** Penetration resistance curves. **b** Normalized penetration resistance curves.

Two-dimensional DEM simulations of static cone penetration tests were conducted under the three gravitational acceleration levels: 1/6 g, 1 g, and 2 g. To account for computational limitations, the numerical model was simplified by reducing domain size, narrowing particle size distribution, and simplifying particle morphology. The penetration resistance and normalized penetration resistance curves are presented in Fig. 2.

A comparison between Fig. 1 and Fig. 2 shows that the trends of penetration resistance and normalized resistance obtained from the DEM simulations are in good agreement with the experimental results. In both cases, the penetration resistance decreases with decreasing gravitational acceleration level, while the normalized penetration resistance increases sharply as gravity decreases. In the DEM simulations, penetration resistance initially increases approximately linearly with depth. Then it reaches a turning point beyond which the growth rate slows—similar to the behavior observed in physical tests at relative densities of 40% and 58%. Moreover, in both DEM simulations and physical tests, the penetration resistance curves corresponding to the three gravitational acceleration levels overlap during the initial stage. Overall, the DEM results qualitatively reproduce the penetration resistance behavior observed in the physical experiments. In terms of the maximum normalized penetration resistance, the DEM simulation results fall between those obtained for relative densities of 40% and 58% in the laboratory tests.

## Discussion

**Fig. 3:**
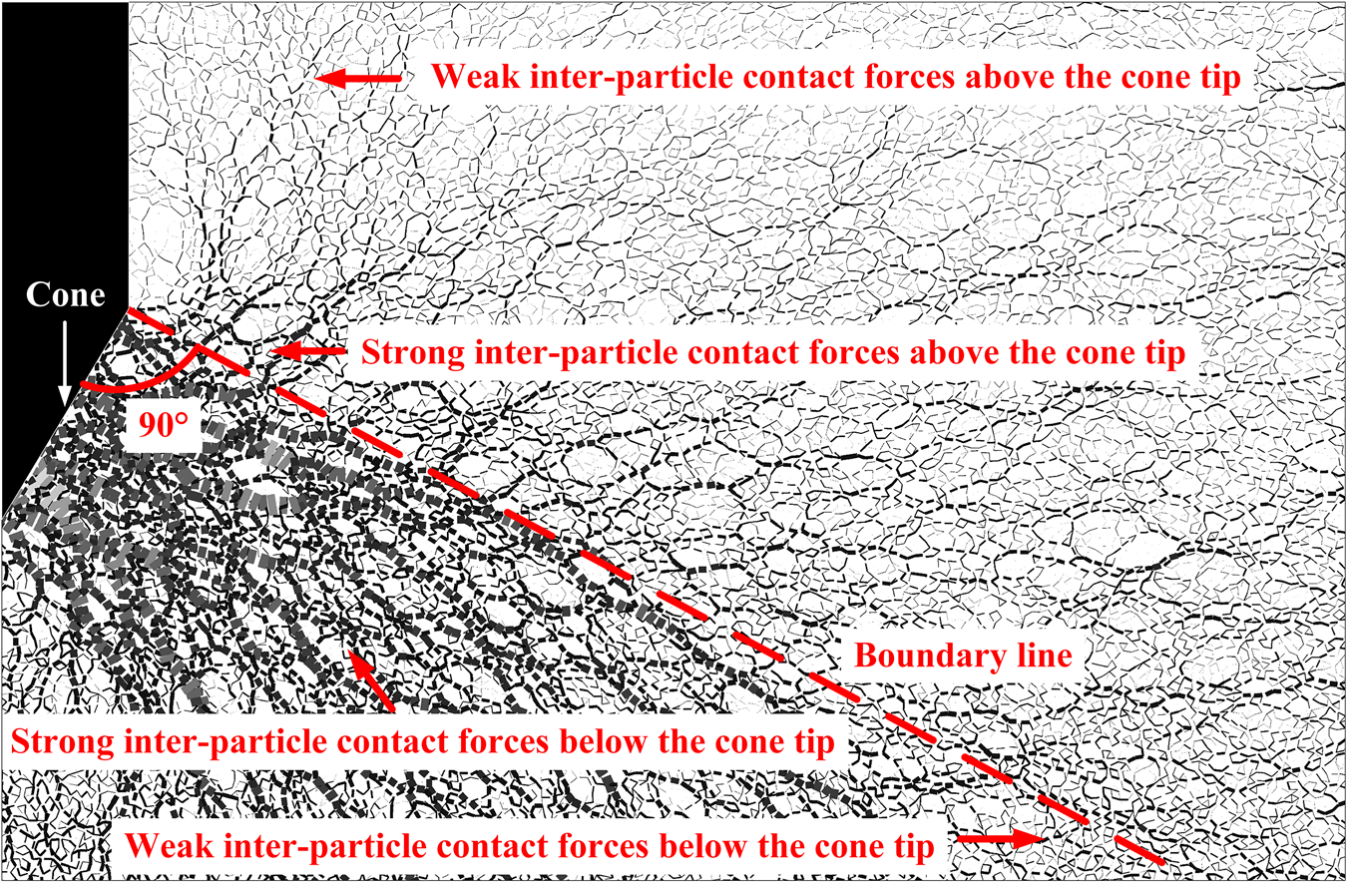
Schematic diagram of classification for inter-particle contact force near the cone tip during penetration.

To interpret the penetration behavior observed in experiments, the inter-particle contact force is used to quantify the mesoscopic influence of gravity on penetration resistance. The extrusion force generated by the cone tip is transmitted through these inter-particle contact forces to the surrounding particles, as illustrated in Fig. 3. For analytical clarity, the inter-particle contact forces are first classified as follows.

Inter-particle contact forces can be classified as strong or weak, with strong forces exceeding the average inter-particle contact force and weak forces falling below it. During the penetration process, the influence of the cone tip resistance is spatially limited, and most contact forces outside this zone are weak, showing only limited correlation with the penetration resistance. Distinguishing between strong and weak inter-particle contact forces enables the identification and isolation of the strong contact forces that are most closely associated with cone tip resistance.

A boundary line is drawn from the upper end of the cone surface along its normal direction, dividing the model domain into upper and lower regions, where the inter-particle contact forces are accordingly defined as upper and lower contact forces. In the absence of gravity, stable force chains cannot form above this boundary, while particles below it can still develop force chains through interlocking and frictional interactions.

**Fig. 4:**
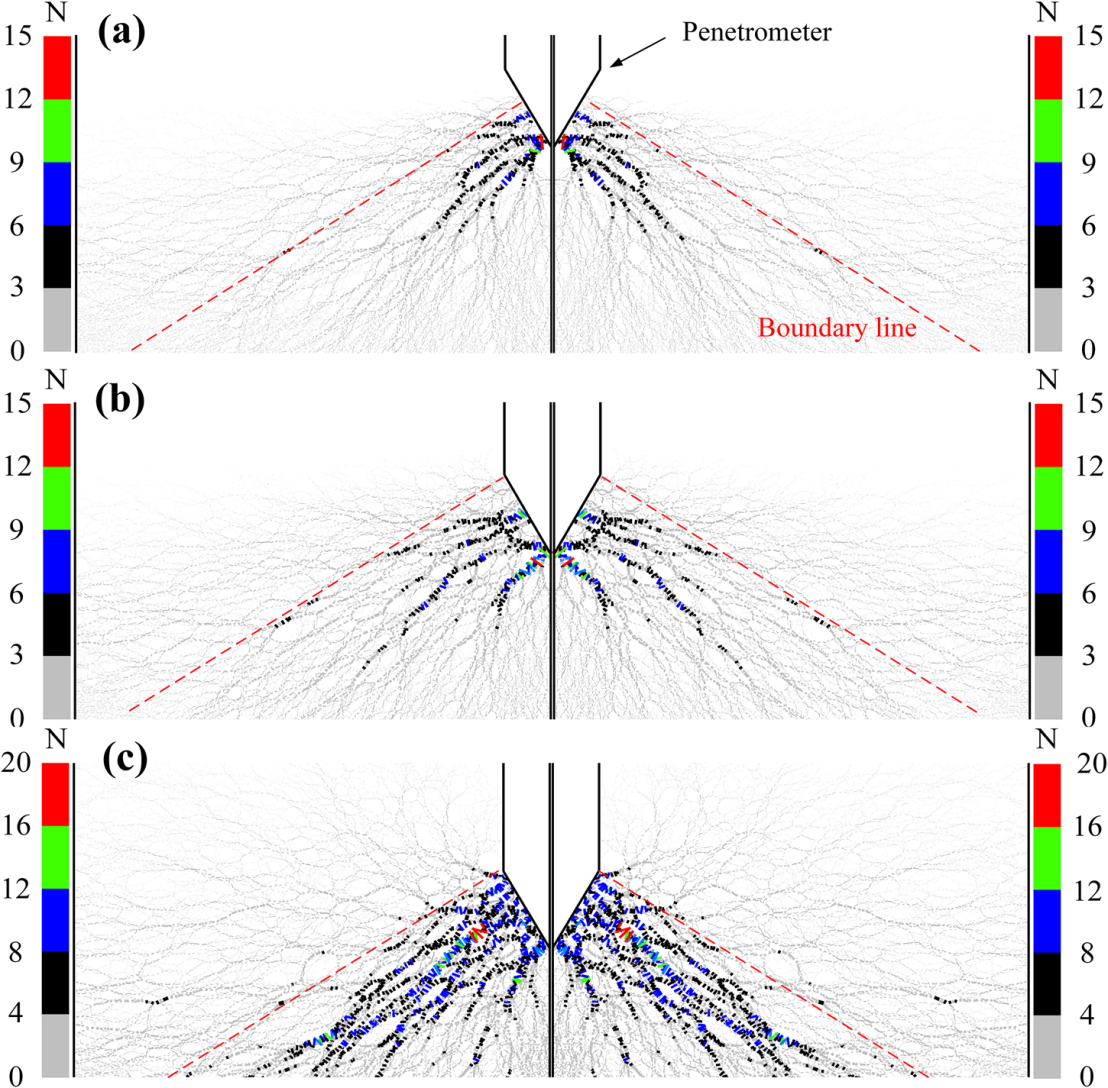
Force chain distribution near the cone tip of CUMT-1 lunar regolith simulant at different penetration stages (DEM simulations). **a** Stage I: Penetration depth = 1.73 mm. **b** Stage II: Penetration depth = 3.47 mm. **c** Stage III: Penetration depth = 10.40 mm.

Taking the 1/6 g gravity condition as an example, Fig. 4 presents the force chain distributions of the CUMT-1 lunar regolith simulant at three representative penetration depths. The thickness and color of each force chain indicate the relative magnitude of the inter-particle contact force. According to the above classification criteria, all force chains except those shown in gray represent strong force chains.

Rapid accumulation stage of the force chain skeleton. Figure 4a shows the distribution of force chains when the cone tip has penetrated to half of its length. At this stage, the influence of vertical stress from the overlying soil is relatively weak, and the resistance generated within the CUMT-1 simulant primarily arises from interlocking and frictional contacts among the underlying particles. This is evidenced by the concentration of strong force chains beneath the cone tip, which tend to propagate along the normal direction of the cone surface. This distribution pattern indicates that even under very low vertical stress, a force chain network capable of resisting downward penetration can form within the simulant. This also explains why the penetration resistance curves under different gravity conditions overlap during the early stage, as shown in Figs. 1a–c and 2a. During this stage, the contact area between the cone surface and the surrounding particles increases with penetration depth, leading to a rapid accumulation of supporting points for strong force chains. This is reflected in Fig. 2a as an approximately linear increase in cone tip resistance, with the highest growth rate observed throughout the entire penetration process.

Completion stage of force chain skeleton development. Figure 4b shows the distribution of force chains when the cone tip becomes fully embedded in the soil. Compared to the initial stage, the transmission distance of strong force chains increases significantly. The supporting points of force chains extend from the cone tip to the midsection of the cone surface, while the upper part of the cone is primarily supported by weak contacts. At this point, the force chain skeleton is nearly fully developed. The rate of increase in penetration resistance begins to decline after this stage, as shown in Fig. 2a.

Continuous strengthening stage of the force chain skeleton. Figure 4c shows the force chain distribution when the cone tip has penetrated to a depth of 10.4 mm, approximately three times the cone height. At this stage, the penetration force transmission pattern has stabilized, and further increases in depth no longer cause significant changes to the primary force chain skeleton surrounding the cone. Gravity becomes the dominant factor contributing to the increase in tip resistance. However, its effect does not occur through the direct formation of strong force chains. Instead, gravity enhances auxiliary force chains above the boundary line, which in turn support the strong force chains oriented along the cone’s normal direction, thereby indirectly increasing the cone tip resistance. As a result, the sensitivity of tip resistance to penetration depth is significantly lower than during the initial rapid accumulation phase of the force chain skeleton.

**Fig. 5:**
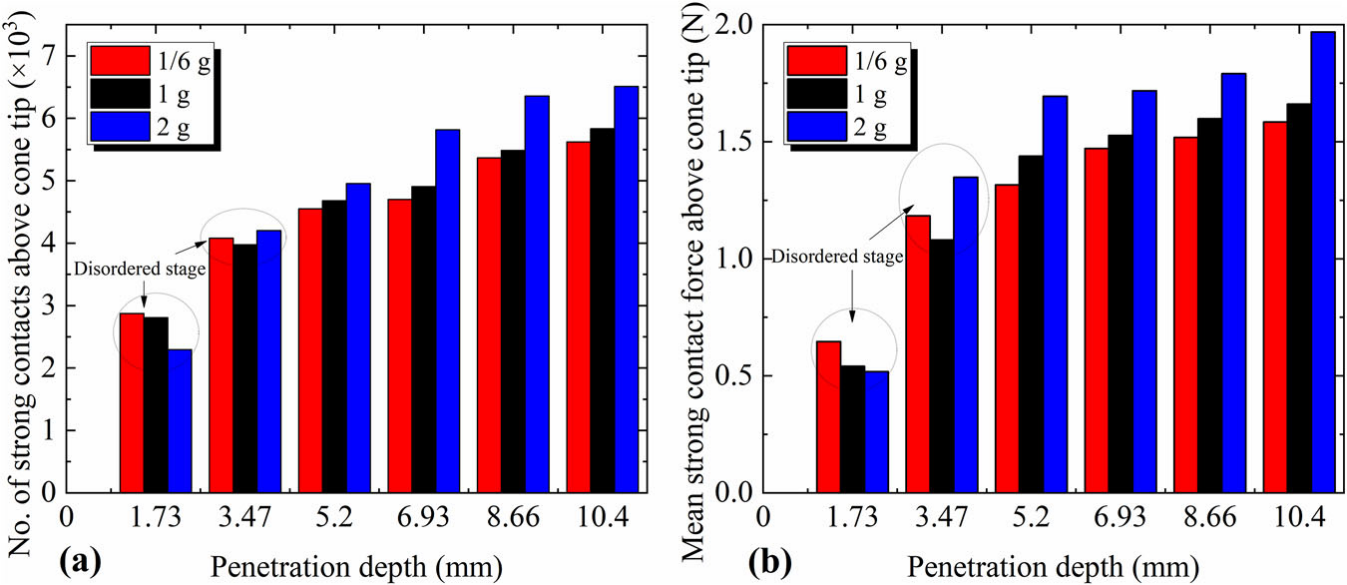
Variation of the number and mean magnitude of strong contacts above the cone tip with penetration depth. **a** Number of strong contacts. **b** Mean magnitude of strong contacts.

Based on the above analysis of the penetration process under 1/6 g gravity, the evolution of a single penetration resistance curve can be interpreted from a microscale perspective. Nevertheless, this factor alone is insufficient to explain the increasing trend of normalized penetration resistance under reduced gravity. As discussed earlier, gravity enhances the cone tip resistance primarily by strengthening auxiliary force chains above the cone tip, which indirectly support the main load-bearing force chains along the cone. Therefore, the subsequent analysis focuses on the strong force chains above the cone tip. Two parameters were selected to characterize the state of these force chains: the number of strong inter-particle contacts above the cone tip, and the average magnitude of these strong inter-particle contact forces. The variations of these two parameters with penetration depth under the three gravitational acceleration conditions are summarized in Fig. 5.

At the initial stage of penetration, the number of strong contacts and the average strong contact force in the upper region of the cone tip show significant overlap across the three gravity conditions. This can be attributed to the rapid accumulation of supporting points for strong force chains along the cone surface at this stage, during which the influence of gravity is relatively minor, as shown in Fig. 4a. To ensure a valid comparison, the stable penetration interval below 5 mm depth was selected for analysis. As shown in Fig. 5a, b, both the number of strong contacts and the mean strong contact force in the upper cone region decrease with lower gravity, which aligns with expectations. However, the magnitude of reduction in these two metrics is considerably smaller than the reduction in gravitational loading. Specifically, when gravity decreases from 2 g to 1/6 g (a 91.67% reduction), the number of strong contacts and the average strong contact force decrease by only ~14.36% and 17.87%, respectively.

The formation of strong force chains above the cone tip is influenced by both vertical stress and the interlocking and frictional structures between particles. At the same penetration depth under 1/6 g and 2 g conditions, although the vertical stress in the latter is twelve times that in the former, the quantity and strength of inter-particle interlocking and frictional structures remain largely comparable. As a result, the penetration resistance under 1/6 g is far less than one-twelfth of that under 2 g, resulting in a substantially higher normalized penetration resistance in the lower gravitational acceleration level. This finding also explains the observed reduction in the gravity sensitivity of normalized penetration resistance under high relative densities, as shown in Fig. 1a–c. Specifically, the number and strength of interlocking and frictional structures above the cone tip increase with increasing relative density, thereby diminishing the relative contribution of gravity to the formation of strong force chains in this region.

By combining GMMT and DEM methods, this study revealed how gravity influences the penetration resistance of CUMT-1 lunar regolith simulant under 1/6 g, 1 g, and 2 g conditions. The results indicate that penetration resistance is primarily governed by two factors: gravitational stress and the interlocking and frictional interactions among particles above the cone tip. These two factors jointly determine the number and strength of strong contacts in the upper region of the cone, which in turn indirectly affect the cone tip resistance. The interlocking and frictional structures are influenced by particle morphology (e.g., roughness, angularity, and aspect ratio) and relative density. Given that in situ lunar regolith exhibits complex particle morphologies and high relative density, it can be inferred that the contribution of interlocking and frictional structures to penetration resistance is likely greater than that in conventional terrestrial soils. Consequently, the sensitivity of cone tip resistance to gravity is expected to be relatively low.

This study suggests that under practical conditions where the equipment weight on the Moon is reduced to one-sixth of that on Earth, the decrease in penetration resistance is likely to be slight. This means that insufficient self-weight support force may become a major obstacle to penetration or drilling operations. Given that the CUMT-1 simulant exhibits irregular particle shapes and high single-particle strength, the penetration resistance data under 1/6 g can be regarded as approximately representative of real lunar regolith. At a relative density of 76%, penetrating a 150 mm layer of CUMT-1 simulant requires a resistance of ~11 MPa, corresponding to a force of about 553 N. This indicates that a lunar rover would need a mass of at least ~342 kg on the Moon to directly penetrate a 150 mm layer of lunar regolith. However, considering that the lunar rover may become unstable if its support points are misaligned, and the possible presence of heterogeneous layers during penetration, the actual required mass would need to be considerably higher. This also helps explain why the Lunokhod 1 and 2 missions conducted nearly 1000 cone penetration tests, each reaching only about 4.4 cm depth, without further penetration.

From an engineering perspective, achieving deeper penetration can be facilitated by three strategies: reducing the drill bit diameter while maintaining sufficient strength; employing an alternative drilling approach, such as a mole-type (self-penetrating) system, rather than direct penetration; and enhancing the lunar rover’s traction or anchoring capability to increase the effective support force.

## Methods

### CUMT-1 lunar regolith simulant

**Fig. 6:**
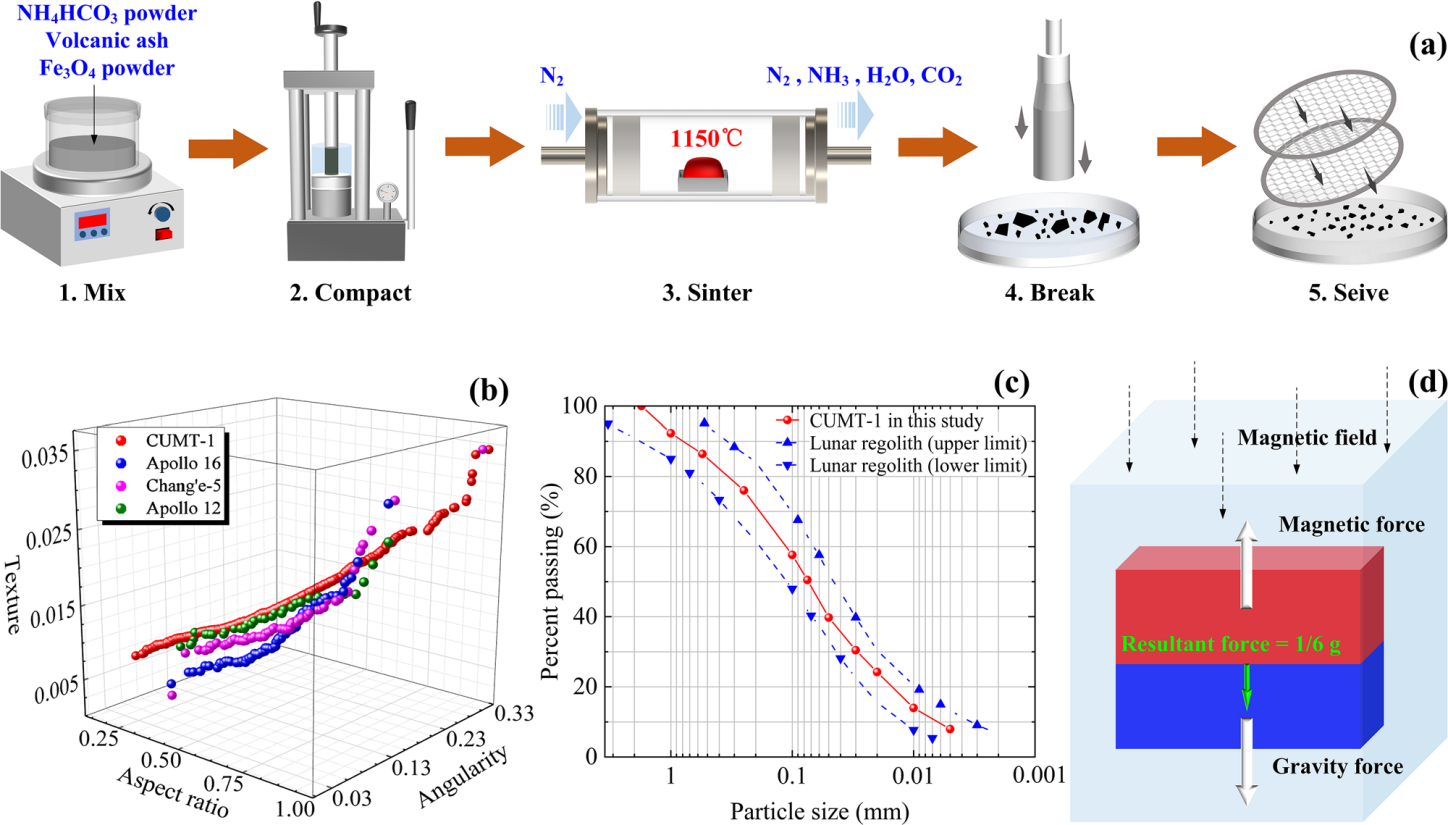
Introduction of the magnetic CUMT-1 lunar regolith simulant. **a** Development process of CUMT-1 lunar regolith simulant. **b** Force state of a CUMT-1 lunar regolith simulant particle in the magnetic field. **c** Particle size distributions of CUMT-1 lunar regolith simulant and lunar surface regolith. **d** Particle morphology distributions of CUMT-1 lunar regolith simulant and lunar surface regolith.

The main procedures for preparing the CUMT-1 lunar regolith simulant, as illustrated in Fig. 6a, are as follows. Volcanic ash and Fe_3_O_4_ magnetic powder were first mixed at a mass ratio of 3:2 using an automatic mixer, after which the mixture was blended with NH_4_HCO_3_ powder at a mass ratio of 4:1. The resulting blend was then compacted under 11 MPa for 90 min using a high-pressure consolidator to form cylindrical specimens, which were carefully demolded to avoid cracking. These compacted specimens were subsequently sintered in a high-temperature tube furnace to produce lunar bedrock simulants, with nitrogen gas continuously supplied during sintering to prevent oxidation of the magnetic powder. The sintered bedrock was crushed using a pneumatic hammer with an impulse of approximately 30 kg·m/s and an operating air pressure of 0.5–0.7 MPa to generate granular regolith particles. Finally, the particles were sieved using a vibrating screen, classified by size, oven-dried, and sealed for later use.

The resulting CUMT-1 simulant particles exhibit morphological features such as surface roughness, angularity, and aspect ratio that closely resemble those of real lunar regolith, as shown in Fig. 6b. The particle size distribution used in this study was based on the average shallow lunar soil gradation summarized by Carrier et al.^44^, as shown in Fig. 6c. The force state of the CUMT-1 lunar regolith simulant in the magnetic levitation apparatus is depicted in Fig. 6d.

### Magnetic levitation system

**Fig. 7:**
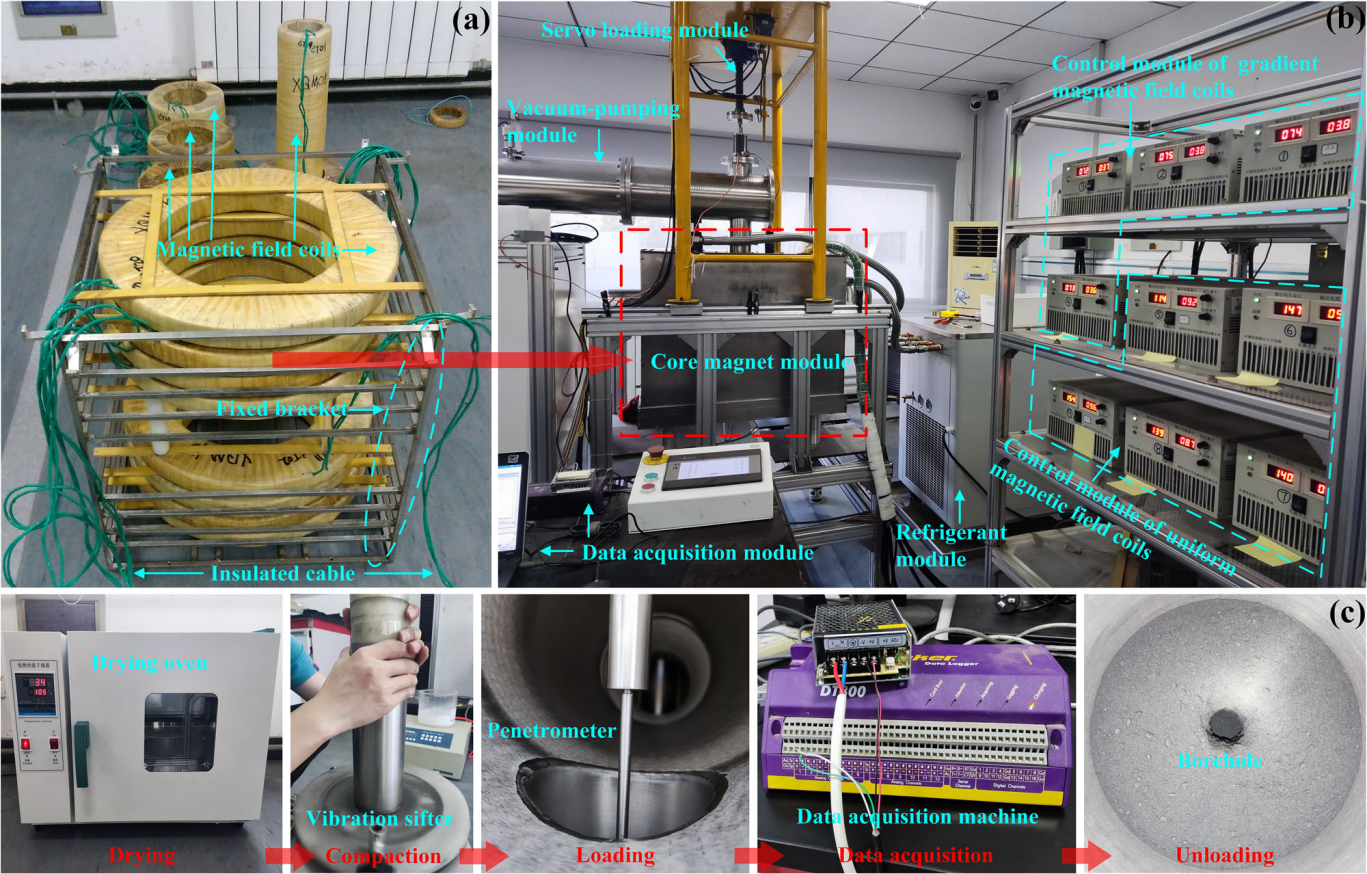
Experimental equipment and procedure of the GMMT method. **a** Cone penetration test process of CUMT-1 lunar regolith simulant. **b** The magnetic levitation system in operational status. **c** Magnetic field coils and their fixed brackets.

The cone penetration tests were conducted using the GMMT method^41^. This approach is implemented based on a magnetic levitation system, whose core components include five sets of uniform magnetic field coils and eight sets of Helmholtz coils, with all coils wound using copper wire, as illustrated in Fig. 7a. The coil assemblies are fixed within a 316 L stainless steel frame and integrated into a sealed cubic chamber, as shown in Fig. 7b. The center of the cube provides a working volume of 95 mm diameter and 160 mm height. During penetration tests at different gravitational acceleration levels, a uniform magnetic field is generated by controlling the current in the uniform field coils, ensuring the CUMT-1 simulant reaches a saturated magnetization state. Subsequently, a magnetic field gradient is induced by controlling the current in the gradient coils, generating an upward magnetic force for the CUMT-1 lunar regolith simulant that counteracts 5/6 of Earth’s gravity.

Significant Joule heating occurs due to the electrical resistance of the copper coils. To manage this heat, thermally conductive silicone oil, chosen for its chemical stability, is circulated through the system. The silicone oil absorbs heat from the coils and is subsequently cooled using a 10 kW chiller. Above the magnetic coil assembly, a servo-driven loading device is mounted to drive the cone tip into the soil at controlled speeds ranging from 0.01 mm/s to 10 mm/s. A servo-driven loading device is mounted above the coil assembly to drive the cone tip into the soil. A force sensor with 800 N capacity is installed at the junction between the cone tip and the servo loading rod to measure penetration resistance. Data from the sensor is recorded using a dataTaker DT800 data acquisition system, as presented in Fig. 7c.

### Accuracy evaluation of 1/6 g gravity simulation

The evaluation process consists of two parts: determining the specific saturation magnetization and assessing the accuracy of the 1/6 g gravity simulation. To measure the specific saturation magnetization of the CUMT-1 lunar regolith simulant, its force state under a magnetic field was first analyzed.

The combined force of magnetic and gravitational forces is called magnetically simulated gravity. Accordingly, the magnetically simulated gravity acting on the CUMT-1 lunar regolith simulant can be expressed as:

4 $${G}^{m}=mg+{\mu }_{0}m{\vartheta }_{s}\frac{\partial {\boldsymbol{H}}}{\partial z}$$

where *G* ^m^ represents the resultant force of gravity and magnetic force, *m* is the mass of the CUMT-1 lunar regolith simulant, *g* is the gravitational acceleration, *μ*_0_ is the vacuum magnetic permeability, *ϑ*_s_ is the specific magnetization under a given magnetic field condition, and $\partial {\boldsymbol{H}}/\partial z$ is the magnetic field gradient intensity. Accordingly, the specific saturation magnetization can be expressed as:

5 $${\vartheta }_{s}=\frac{{G}^{m}-mg}{{\mu }_{0}m\frac{\partial {\boldsymbol{H}}}{\partial z}}$$

**Fig. 8:**
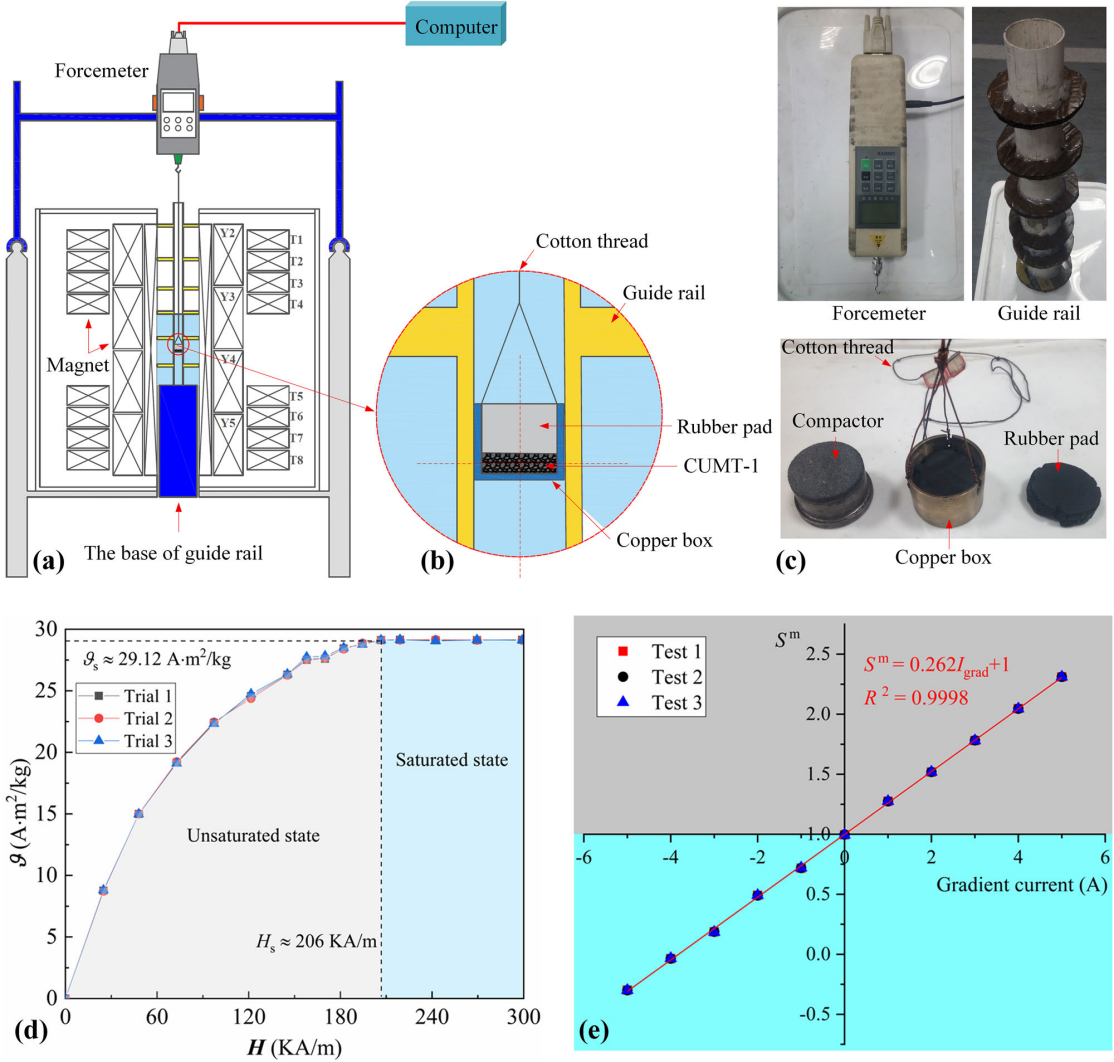
Equipment and results for 1/6 g gravity accuracy evaluation. **a** Cross-section of the test equipment. **b** Enlarged schematic of the test container. **c** Physical view of key test components. **d** Specific saturation magnetization curve of CUMT-1 lunar regolith simulant. **e** Magnetic-gravity characteristic curve of CUMT-1 lunar regolith simulant.

The specific saturation magnetization can be calculated according to Eq. (5). Specifically, a 100 g sample was placed in a copper cylinder sealed with a plastic lid. The cylinder was connected to a force gauge using a cotton thread and suspended within the effective test zone, as shown in Fig. 8a–c. A gradient magnetic field of 110 kA/m was first applied, followed by a gradual increase in the uniform magnetic field while monitoring the force gauge. When the readings of the force gauge no longer changed appreciably between successive measurements, the material was considered to have reached magnetic saturation. Based on this procedure, the specific saturation magnetization curve of the CUMT-1 lunar regolith simulant was shown in Fig. 8d, with a maximum value of 29.12 A·m^2^/kg.

To further evaluate the accuracy of the magnetic-gravity field simulation, the uniform magnetic field intensity was maintained at 32 A·m^2^/kg to ensure that the CUMT-1 lunar regolith simulant was fully magnetized. The gradient magnetic field was then gradually increased, and the resultant force of gravity and magnetic forces acting on the CUMT-1 simulant under different gradient field conditions was recorded to assess the deviation between the theoretical magnetic-gravity field and the measured values. For clarity in illustrating the accuracy of the magnetic-gravity field simulation, both sides of Eq. (4) were divided by *mg*, yielding the following expression.

6 $${S}^{m}=\frac{{g}_{m}}{g}=1+\frac{{\mu }_{0}{\vartheta }_{s}}{g}\frac{\partial {\boldsymbol{H}}}{\partial z}$$

where *S* ^m^ is defined as the magnetic-gravity field similarity constant, with values greater than, equal to, and less than 1 indicating that the material is under hypergravity, normal gravity, and microgravity conditions, respectively.

According to Eq. (6), the magnetic-gravity field similarity constant is linearly related to the gradient magnetic field intensity, which is proportional to the current in the gradient magnet. Therefore, in practical evaluations, the linearity between the magnetic-gravity field similarity constant and the current can be directly observed to assess the accuracy of the magnetic-gravity field simulation. The relationship between the magnetic-gravity field similarity constant and the current is shown in Fig. 8e. The R^2^ value of 0.9998 indicates a strong linear correlation, confirming the high accuracy of the magnetic-gravity field simulation.

### Analysis of interparticle attraction induced by magnetic forces

Under the influence of a magnetic field, the CUMT-1 lunar regolith simulant particles experience not only an upward magnetic force that counteracts gravity, but also a distance-dependent interparticle attraction. From a geotechnical perspective, this magnetic attraction can be interpreted as an apparent cohesion. Therefore, when analyzing the experimental results, it is crucial to first assess the potential impact of this additional attractive force on the mechanical strength of the simulant.

**Fig. 9:**
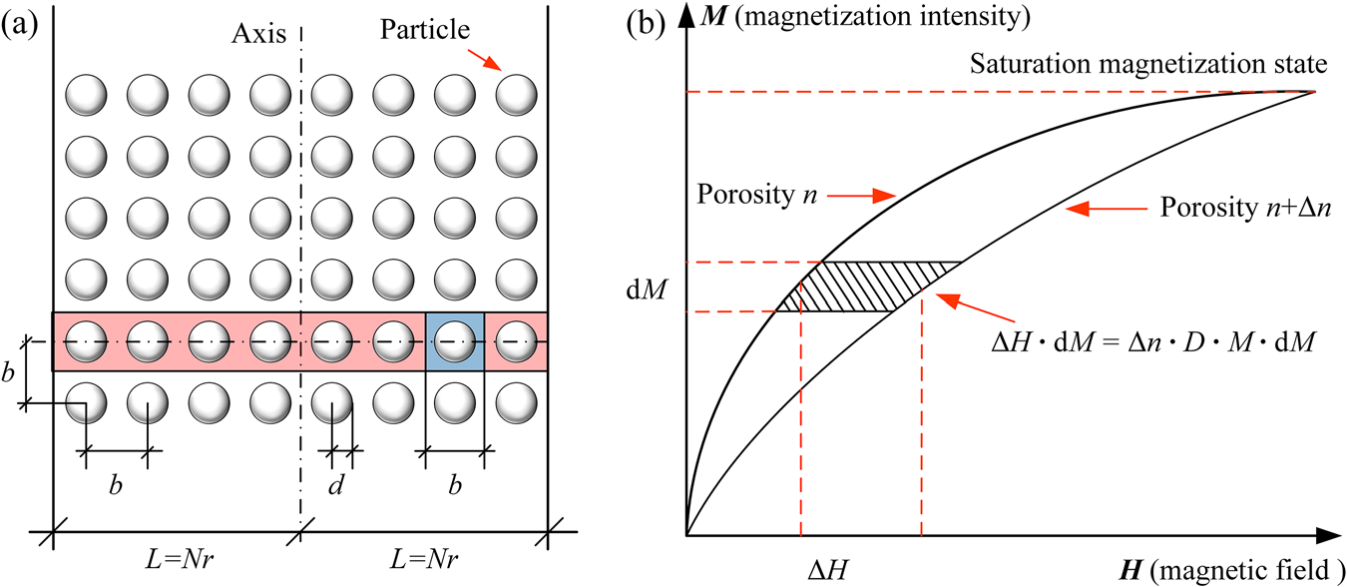
Calculation model of interparticle attraction induced by magnetic forces. **a** Schematic diagram of interparticle attraction induced by magnetic forces. **b** Variation of single particle energy storage with porosity.

For analytical purposes, the particles are assumed to be spherical and uniformly arranged within an infinitely long domain of width 2 *L*, as illustrated in Fig. 9a. The particle assembly is characterized by a porosity *n*, particle radius *d*, and interparticle spacing *b*.

A red rectangular representative element is selected from Fig. 9a for analysis. The element is assumed to have a thickness of *b* (note that only the projection plane is shown in the schematic), a length of *2* *L*, and contains *N* particles. The porosity *n* of the representative element can be calculated as follows:

7 $$n=\frac{V{\rm{porous}}}{V{\rm{total}}}=\frac{V{\rm{total}}-V{\rm{solid}}}{V{\rm{total}}}$$

where *V*_total_, *V*_porous_, and *V*_solid_ represent the total volume of the red representative element, the pore volume, and the particle volume, respectively. By rearranging Eq. (7) and incorporating the geometric relationships of the particles within the model, the following expression is obtained:

8 $$1-n=\frac{V{\rm{solid}}}{V{\rm{total}}}=\frac{4\pi {d}^{3}}{3{b}^{3}}$$

After further simplification, the relationship between the interparticle spacing and the particle radius can be expressed as:

9 $$b=d{(\frac{4\pi }{3(1-n)})}^{1/3}$$

When being magnetized in a magnetic field ***H***, the magnetic attraction between particles is denoted as ${\tilde{F}}^{{\rm{m}}}$. Given that the magnetic force is inversely proportional to the fourth power of the interparticle distance^45^, only the interaction with the nearest neighboring particles is considered.

Assuming that the particle assembly deforms under magnetic forces, resulting in a change in porosity from *n* to *n* + Δ*n*, a corresponding change in stored energy Δ*W*, and a total displacement of the assembly Δ*u*, the following relationship can be established:

10 $$\varDelta W={\tilde{F}}^{{\rm{m}}}\varDelta u=2N\cdot \delta W$$

where *W* represents the stored energy per particle. The total displacement Δ*u* can be expressed as:

11 $$\Delta u=2N\Delta b=2N\frac{{\rm{d}}}{{\rm{d}}n}\left(d{\left(\frac{4\pi }{3(1-n)}\right)}^{1/3}\right)=\frac{2dN}{3}{\left(\frac{4\pi }{3}\right)}^{1/3}\frac{\Delta n}{{(1-n)}^{4/3}}$$

Figure 9b presents the magnetization curve of the representative element, along with the variation in the curve resulting from changes in porosity. It can be observed that the change in energy stored by a single particle, induced by the porosity variation, corresponds to the shaded area between the two magnetization curves. Therefore, the energy stored per particle can be expressed as:

12 $$\delta W=V\int \varDelta H{\rm{d}}M$$

where *V* denotes the volume of a single particle.

According to the principles of magnetic material magnetization, the effective magnetic field *H*_eff_ acting within a magnetic material is given by the difference between the externally applied magnetic field and the demagnetizing field generated within the particle:

13 $${H}_{\mathrm{eff}}=H-{\chi }^{{\rm{m}}}nM$$

where ${\chi }^{{\rm{m}}}$ is the demagnetization factor, which depends solely on the particle geometry.

Therefore, the change in magnetic field intensity experienced by a single particle due to the variation in porosity can be expressed as:

14 $$\varDelta H=\varDelta n{\chi }^{{\rm{m}}}nM$$

Substituting the above expression into Eq. (12) yields:

15 $$\delta W=V\int \varDelta n{\chi }^{{\rm{m}}}M{\rm{d}}M=\frac{2\pi }{3}{d}^{3}\varDelta n{\chi }^{{\rm{m}}}{M}^{2}$$

Substituting the above expression into Eq. (10) yields:

16 $${\tilde{F}}^{{\rm{m}}}=\frac{4\pi }{3}\frac{{d}^{3}\varDelta nN{{\rm{\chi }}}^{{\rm{m}}}{M}^{2}}{\varDelta u}$$

Further substituting the above expression into Eq. (11) yields:

17 $${\tilde{F}}^{{\rm{m}}}{=(6{\rm{\pi }}}^{2}{)}^{1/3}{d}^{2}{(1-n)}^{4/3}{{\rm{\chi }}}^{m}{M}^{2}$$

During all the experiments, CUMT-1 particles were in a saturated magnetic state. Therefore, interparticle attraction induced by magnetic forces in the above equation can be expressed in terms of the specific saturation magnetization as follows:

18 $${\tilde{F}}^{{\rm{m}}}{=(6{\rm{\pi }}}^{2}{)}^{1/3}{{\rm{\chi }}}^{m}{\rho }^{2}{d}^{2}{(1-n)}^{4/3}{({\vartheta }_{{\rm{s}}})}^{2}$$

where *ρ* denotes the bulk density of the particle assembly, and *ϑ*_s_ represents the specific saturation magnetization of the CUMT-1 lunar regolith simulant. Accordingly, the interparticle attraction induced by magnetic forces *c*_m_ can be expressed as:

19 $${c}_{{\rm{m}}}=\frac{{\tilde{F}}^{{\rm{m}}}}{{d}^{2}}{=(6{\rm{\pi }}}^{2}{)}^{1/3}{{\rm{\chi }}}^{m}{\rho }^{2}{(1-n)}^{4/3}{({\vartheta }_{{\rm{s}}})}^{2}$$

By further considering the relationship between the bulk density of the particle assembly and porosity as $\rho =(1-n){\rho }_{{\rm{s}}}$, Eq. (19) can be ultimately expressed in the following form:

20 $${c}_{{\rm{m}}}=\frac{{\tilde{F}}^{{\rm{m}}}}{{d}^{2}}{=(6{\rm{\pi }}}^{2}{)}^{1/3}{{\rm{\chi }}}^{m}{(1-n)}^{10/3}{({\rho }_{{\rm{s}}}{\vartheta }_{{\rm{s}}})}^{2}$$

where ${\rho }_{s}$ denotes the specific gravity of the CUMT-1 lunar regolith simulant.

**Fig. 10:**
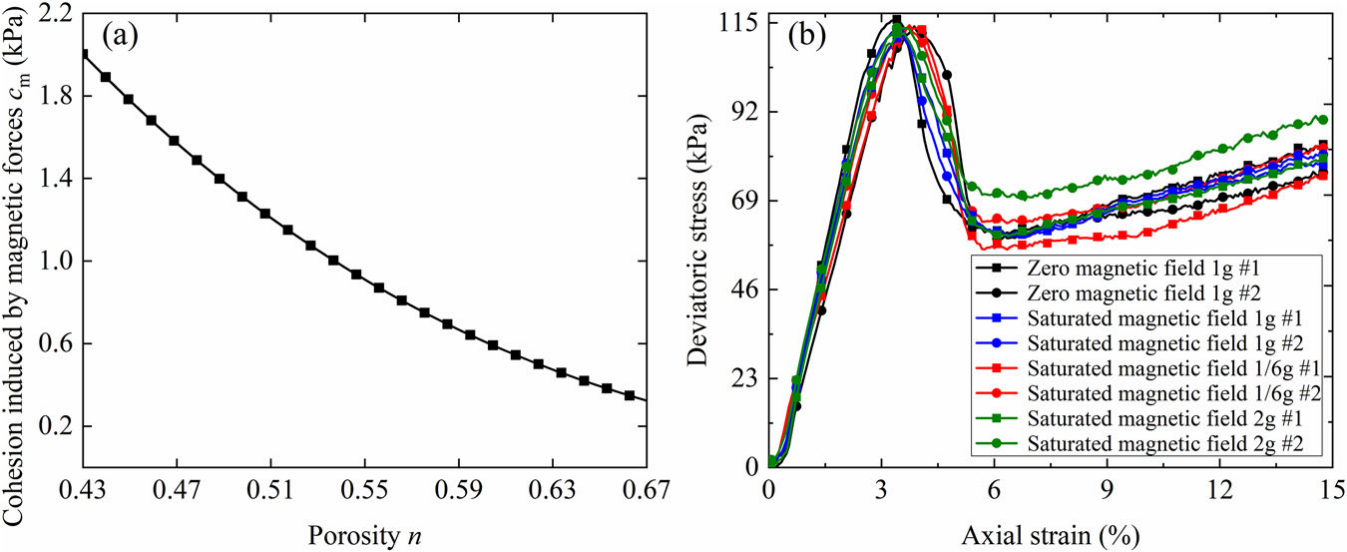
Estimated range of magnetically induced apparent cohesion in CUMT-1 lunar regolith simulant and its effect on macroscopic strength. **a** Relationship between porosity and magnetically induced cohesion. **b** Triaxial stress-strain behaviors under varying magnetic fields.

For the given CUMT-1 lunar regolith simulant, the demagnetization factor, specific gravity, and specific saturation magnetization are constants. Consequently, the interparticle attraction induced by magnetic forces depends solely on porosity, and the corresponding relationship is illustrated in Fig. 10a.

Except for the assumption of spherical particle geometry, all other parameters used in the calculations were obtained from experimental measurements: the demagnetization factor was taken as 1/3, the specific gravity as 3.44, and the specific saturation magnetization as 29.12 A·m²/kg. It can be seen that the magnetically induced apparent cohesion of the CUMT-1 lunar regolith simulant decreases with increasing porosity. The maximum relative density for CUMT-1 simulant in this study reached 76%, corresponding to a peak additional cohesion of less than 2 kPa.

To further quantify the effect of magnetically induced apparent cohesion, triaxial tests were performed on CUMT-1 lunar regolith simulant at a relative density of 74% under varying magnetic fields, with a low confining pressure of 10 kPa. The triaxial test was chosen because it is a well-established method in geotechnical engineering for evaluating the mechanical strength of granular materials. The internal friction angle obtained from triaxial tests can be directly used to predict static penetration resistance. For instance, Uzielli et al.^46^ demonstrated a strong correlation between the internal friction angle and penetration resistance data. Hence, the triaxial test provides a reliable and quantitative basis for assessing the sensitivity of the CUMT-1 simulant’s mechanical response to magnetically induced apparent cohesion.

The results are shown in Fig. 10b. It can be observed that the stress-strain behavior under the magnetic field is close to that in a non-magnetic (Earth-like) environment. Moreover, the fluctuations observed in the stress-strain curves across different magnetic conditions are of the same order of magnitude as those observed in parallel tests conducted under the same magnetic field. Therefore, it can be concluded that the magnetically induced apparent cohesion has a negligible effect on the strength of the CUMT-1 lunar regolith simulant.

### Physical cone penetration test method

To replicate the absolute dryness of lunar regolith in laboratory conditions, the CUMT-1 lunar regolith simulant was oven-dried at 105 °C for 8–12 h before each test, as shown in Fig. 7c. After drying, the simulant was sealed in plastic film and stored until it cooled to room temperature for subsequent use. Regarding the model dimensions, the cone penetration rod had a diameter of 6 mm, a cone angle of 60°, and a base diameter of 8 mm. The model container was 250 mm in depth, with an internal diameter of 81 mm. In terms of penetration velocity, a rate of 0.55 mm/s was employed, corresponding to approximately 1.83% of the typical speed used in parabolic-flight experiments^26,27^. This low-speed setting more closely reflects the working conditions of cone penetration on the Moon. Nevertheless, 0.55 mm/s is the maximum speed in the current setup due to copper magnet thermal limits and may still not fully satisfy quasi-static conditions. Thus, dynamic effects cannot be ruled out entirely, and the results should therefore be interpreted with caution.

Cone penetration resistance is sensitive to the degree of compaction in the simulant, and sample uniformity plays a critical role in ensuring the reliability of test results. The commonly used layered under-compaction method proved inadequate for achieving uniform density in CUMT-1, especially at high compaction conditions, where density always increases significantly with depth. After extensive trial and error, a vibration-based compaction method was adopted. This approach yielded samples with relatively uniform density across depths. The equipment used for this method is shown in Fig. 7c. For samples with specified mass and target relative density, the required compaction height was first calculated, and the corresponding position was marked on the epoxy resin mold. The simulant was then carefully funneled into the mold using a long-neck funnel, and the surface was gently leveled and compressed with a mold cap. The entire mold was subsequently pressed against a vibratory sieve shaker, and high-frequency vibration was applied until the compacted sample surface reached the predetermined mark, indicating the target compaction had been achieved.

### DEM simulation method

The DEM method has been widely applied to the cone penetration studies of lunar regolith in recent years^47–49^. This method abstracts the mechanical behavior of real-world materials into a set of simplified mathematical models, enabling the investigation of the microscale mechanical responses of numerical particles governed by these models. Consequently, the accuracy and reliability of DEM simulations are critically dependent on the rationality of the model parameters. However, due to current computational limitations, DEM simulations cannot fully replicate the detailed conditions of physical experiments^50,51^. As a result, simplifications are often required in multiple aspects, including spatial dimensions, sample size, particle morphology, particle size distribution, and contact models.

Under these asymmetrical mapping conditions between numerical and physical experiments, it is typically necessary to iteratively calibrate model parameters to reproduce the mechanical behaviors observed in laboratory tests. However, this calibration process is largely empirical and faces three major challenges: microscopic parameters may be tuned to physically unrealistic values that deviate from actual material behavior; the calibrated parameters often lack generalizability and can become invalid with even minor changes in experimental conditions; and substantial discrepancies exist among parameter sets recommended by different researchers for lunar regolith simulants, making it difficult to determine which combination is most appropriate.

At the current stage, it is unrealistic to pursue a universally applicable set of mesoscopic parameters for the CUMT-1 lunar regolith simulant. Therefore, instead of spending significant effort on calibrating micromechanical parameters, this study directly adopted the mesoscopic parameters recommended by Jiang et al.^24^ for the TJ-1 simulant to represent the CUMT-1 simulant, as presented in Table 1. The reason for adopting this approach is that the shear strength obtained using this group of parameters is relatively close to that observed in physical experiments on the CUMT-1 lunar regolith simulant. This consistency may be attributed to the similarity in particle morphology between the CUMT-1 and TJ-1 simulants. It should be noted that the DEM simulations are intended to investigate the qualitative effects of gravity on penetration resistance and to explore the underlying mesoscale mechanisms, rather than to achieve exact numerical agreement with physical experiments. To correspond with the physical experiments, numerical simulations were conducted under three gravitational conditions: 1/6 g, 1 g, and 2 g.

**Table 1:** Input parameters of the DEM cone penetration tests

Parameters	Value	Parameters	Value
Damping ratio, *ζ*	0.7	Particle density, *ρ* (kg/m^3^)	3.44 ×10^3^
Void ratio, *e*	0.06	Particle normal stiffness, *k*_*n*_ (N/m)	7.5 ×10^7^
Wall frictional coefficient, $\mu$_*w*_	0	Particle shear stiffness, *k*_*s*_ (N/m)	5.0 ×10^7^
Particle frictional coefficient, $\mu$_*p*_	0.76	Particle-wall normal stiffness, *k*_*n*_ (N/m)	1.5 ×10^10^
Particle size, *d* (mm)	0.17	Particle-wall shear stiffness, *k*_*s*_ (N/m)	1.0 ×10^10^

**Fig. 11:**
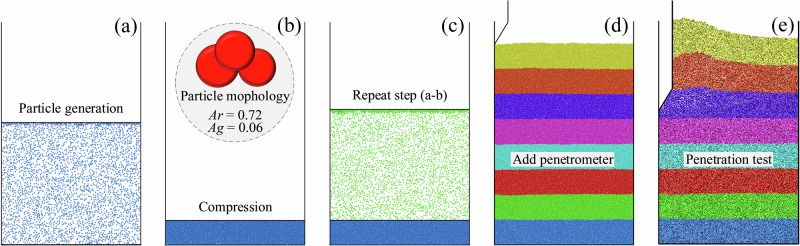
Layered sample preparation method with sawtooth wall. **a** Particle generation. **b** Sample compaction. **c** Cyclic sample preparation. **d** Add penetrometer. **e** Cone penetration.

For other model parameters, the particle shape of the CUMT-1 lunar soil simulant was represented by a clump composed of three overlapping spheres, with an aspect ratio of 0.72 and angularity of 0.06, as shown in Fig. 11b. The particle diameter was uniformly set to 0.17 mm, and the loading velocity was fixed at 5.5 mm/s. The dimensions of the model container were 41 × 30 mm, with both the probe rod and cone having a diameter of 4 mm. Owing to the geometric symmetry of the cone penetration test model, only half of the model was simulated to reduce computational cost. Regarding sample preparation, a layered sample preparation method was adopted. To enhance interlocking between particles at different layers, a sawtooth wall was used instead of a smooth wall. The sample preparation procedure is illustrated in Fig. 11.

### Reporting summary

Further information on research design is available in the Nature Research Reporting Summary linked to this article.

## Data Availability

All the relevant data relating to this study are available upon reasonable request to the co-authors. In addition, the Magnetic Levitation System used in this study can be accessed by contacting the corresponding author. Experimental use of the system is permitted following participation in the required training and approval procedures. All the relevant codes relating to this study are available upon reasonable request to the co-authors.

## Code availability

All the relevant codes relating to this study are available upon reasonable request to the co-authors.

## References

[1] Anttila, M. *Concept Evaluation of Mars Drilling and Sampling Instrument* (Helsinki University of Technology, 2005).

[2] Gao, M. et al. The novel idea and technical progress of lunar in-situ condition preserved coring. *Geomech. Geophys. Geo Energy Geo Resour.* **8**, 46 (2022).

[3] Liang, J. et al. Analysis of the lunar regolith sample obstruction in the Chang’e-5 drill and its improvement. *Adv. Space Res.* **69**, 2248–2258 (2022).

[4] Benaroya, H., Bernold, L. & Chua, K. M. Engineering, design and construction of lunar bases. *J. Aerosp. Eng.* **15**, 33–45 (2002).

[5] Toklu, Y. C. & Akpinar, P. Lunar soils, simulants and lunar construction materials: An overview. *Adv. Space Res.* **70**, 762–779 (2022).

[6] Lee, S. & van Riessen, A. A review on geopolymer technology for lunar base construction. *Materials* **15**, 4516 (2022). 35806640 10.3390/ma15134516 PMC9267385

[7] Ellery, A. Sustainable in-situ resource utilization on the Moon. *Planet. Space Sci.* **184**, 104870 (2020).

[8] Ellery, A. Leveraging in situ resources for lunar base construction. *Can. J. Civ. Eng.* **49**, 657–674 (2022).

[9] Wang, Y. et al. In-situ utilization of regolith resource and future exploration of additive manufacturing for lunar/martian habitats: a review. *Appl. Clay Sci.* **229**, 106673 (2022).

[10] Slyuta, E. N. Physical and mechanical properties of the lunar soil (a review). *Sol. Syst. Res.* **48**, 330–353 (2014).

[11] Qian, Y. et al. The regolith properties of the Chang’e-5 landing region and the ground drilling experiments using lunar regolith simulants. *Icarus* **337**, 113508 (2020).

[12] Jayathilake, B. A. C. S., Ilankoon, I. M. S. K. & Dushyantha, M. N. P. Assessment of significant geotechnical parameters for lunar regolith excavations. *Acta Astronaut.* **196**, 107–122 (2022).

[13] Johnson, S. W. & Chua, K. M. Properties and mechanics of the lunar regolith. *Appl. Mech. Rev.* **46**, 285–300 (1993).

[14] Nie, J. et al. Predicting residual friction angle of lunar regolith based on Chang’e-5 lunar samples. *Sci. Bull.* **68**, 730–739 (2023). 10.1016/j.scib.2023.03.019 36964088

[15] Houston, W. N. & Namiq, L. I. Penetration resistance of lunar soils. *J. Terramech.* **8**, 59–69 (1971).

[16] Robertson, P. K. Interpretation of cone penetration tests—a unified approach. *Can. Geotech. J.* **46**, 1337–1355 (2009).

[17] Heiken, G., Vaniman, D. & French, B. M. (eds) *Lunar Sourcebook: A User’s Guide to the Moon* (Cup Archive, 1991).

[18] Melzer, K. J. Methods for investigating the strength characteristics of a lunar soil simulant. *Geotechnique* **24**, 13–20 (1974).

[19] Rahmatian, L. A. & Metzger, P. T. Soil test apparatus for lunar surfaces. In *Earth and Space 2010: Engineering, Science, Construction, and Operations in Challenging Environments*, 239–253 (American Society of Civil Engineers, 2010).

[20] Cil, M. B. *Discrete Element Modeling of Cone Penetration in JSC-1a Lunar Regolith Simulant*. PhD thesis (Louisiana State Univ. Agric. & Mech. Coll., 2011).

[21] Seweryn, K. et al. Determining the geotechnical properties of planetary regolith using low velocity penetrometers. *Planet. Space Sci.* **99**, 70–83 (2014).

[22] Atkinson, J. et al. Penetration and relaxation behavior of dry lunar regolith simulants. *Icarus* **328**, 82–92 (2019).

[23] Jiang, M. et al. Estimation of mechanical parameters of Tongji-1 lunar soil simulant based on cone penetration test. *Eur. J. Environ. Civ. Eng.* **26**, 393–408 (2022).

[24] Jiang, M., Zhao, T. & Wang, X. DEM modelling of cone penetration tests in lunar soil. *Granul. Matter* **24**, 1–16 (2022).

[25] Sun, Q., Wang, L., Zhang, L., Badal, J. & Chen, Q. Lunar highlands simulant—geotechnical characterization, 3D discrete element modeling, and cone penetration simulations. *Acta Astronaut.* **221**, 283–295 (2024).

[26] Costes, N. C., Cohron, G. T. & Moss, D. C. Cone penetration resistance test—an approach to evaluating in-place strength and packing characteristics of lunar soils. *Proc. Lunar Sci. Conf.* **2**, 1973 (1971).

[27] Daca, A., Tremblay, D. & Skonieczny, K. The relationship between cone penetration resistance and wheel-soil interactions in lunar gravity. In *Proc. IEEE Aerospace Conf*. 1–11 (IEEE, 2021).

[28] Marchese, A. J., Dryer, F. L., Colantonio, R. O. & Nayagam, V. Microgravity combustion of methanol and methanol/water droplets: drop tower experiments and model predictions. *Symp. Int. Combust*. **26**, 1209–1217 (1996).

[29] Sharp, L. M., Dietrich, D. L. & Motil, B. J. Microgravity fluids and combustion research at NASA Glenn Research Center. *J. Aerosp. Eng.* **26**, 439–450 (2013).

[30] Selig, H., Dittus, H. & Lämmerzahl, C. Drop tower microgravity improvement towards the nano-g level for the MICROSCOPE payload tests. *Microgravity Sci. Technol.* **22**, 539–549 (2010).

[31] Kufner, E. et al. ESA’s drop tower utilisation activities 2000 to 2011. *Microgravity Sci. Technol.* **23**, 409–425 (2011).

[32] Sun, P., Wu, C., Zhu, F., Wang, S. & Huang, X. Microgravity combustion of polyethylene droplet in drop tower. *Combust. Flame* **222**, 18–26 (2020).

[33] Liu, T., Wu, Q., Sun, B. & Han, F. Microgravity level measurement of the Beijing drop tower using a sensitive accelerometer. *Sci. Rep.* **6**, 31632 (2016). 27530726 10.1038/srep31632 PMC4987679

[34] Onishi, Y. et al. Observation of flame spreading over electric wire under reduced gravity condition given by parabolic flight and drop tower experiments. *Trans. Jpn. Soc. Aeronaut. Space Sci. Aerosp. Technol. Jpn.* **8**, Ph_19–Ph_24 (2010).

[35] VV, N., Nair, A., P, N., Kumar, A. & TM, M. The 2.5 s microgravity drop tower at National Centre for Combustion Research and Development (NCCRD), Indian Institute of Technology Madras. *Microgravity Sci. Technol.* **30**, 663–673 (2018).

[36] Comstock, D. & Petro, A. Reduced gravity technology demonstration results from NASA’s FAST program and future plans. In *47th AIAA Aerospace Sciences Meeting including The New Horizons Forum and Aerospace Exposition*, 188 (AIAA, 2009).

[37] Hrvatin, E. Biomechanics in weightlessness. *PAJ Perform. Art. J.* **24**, 102–107 (2002).

[38] Kirkpatrick, A. et al. The use of the National Research Council of Canada’s Falcon 20 Research Aircraft as a terrestrial analogue space environment (TASE) for space surgery research: challenges and suggested solutions. *Planet. Space Sci.* **58**, 717–723 (2010).

[39] Geim, A. Everyone’s magnetism: Though it seems counterintuitive, today’s research magnets can easily levitate seemingly nonmagnetic objects, thereby opening an Earthbound door to microgravity conditions. *Phys. Today* **51**, 36–39 (1998).

[40] Sanavandi, H. & Guo, W. A magnetic levitation based low-gravity simulator with an unprecedented large functional volume. *npj Microgravity* **7**, 40 (2021). 34716356 10.1038/s41526-021-00174-4 PMC8556250

[41] Li, R., Zhou, G., Chen, G., Hall, M. R. & Zhao, X. Geotechnical magnetic-similitude-gravity model testing method. *Int. J. Phys. Model. Geotech.* **19**, 181–199 (2019).

[42] Li, R. et al. Preparation and characterization of a specialized lunar regolith simulant for use in lunar low gravity simulation. *Int. J. Min. Sci. Technol.* **32**, 1–15 (2022).

[43] Mitchell, J. K. et al. *Apollo Soil Mechanics Experiment S-200* (California Univ., 1974).

[44] Carrier III, W. D. Particle size distribution of lunar soil. *J. Geotech. Geoenviron. Eng.* **129**, 956–959 (2003).

[45] Eyssa, Y. M. & Boom, R. W. Magnetic and coagulation forces on a suspension of magnetic particles. *Int. J. Miner. Process.* **3**, 1–8 (1976).

[46] Uzielli, M., Mayne, P. W. & Cassidy, M. J. Probabilistic assignment of design strength for sands from in-situ testing data. In *Modern Geotechnical Design Codes of Practice*, 214–227 (IOS Press, 2013).

[47] Wang, L. et al. 3D discrete element modeling of cone penetration into the JSC-1A lunar regolith. *Geo Congr.* **2023**, 172–179 (2023).

[48] Sun, Q. et al. Lunar highlands simulant—geotechnical characterization, 3D discrete element modeling, and cone penetration simulations. *Acta Astronautica.* **221**, 283–295 (2024).

[49] Badal, J., Chen, Q., Zhang, L. & Wang, L. Discrete element modeling of LHS-1 lunar highlands simulant and cone penetrometer–regolith interactions. In *Earth and Space 2024: Engineering for Extreme Environments*, 45–55 (American Society of Civil Engineers, 2024).

[50] Zhao, X., Liu, Z., Li, Y., Wang, H. & Xu, Z. Numerical study of cone penetration tests in lunar regolith for strength index. *Appl. Sci.* **14**, 10645 (2024).

[51] Sanlang, S. et al. 3D discrete element method modeling of the cone penetration test in lunar regolith simulant with various internal friction angles. *Geo. Spatial Inf. Sci*. 1–16, 10.1080/10095020.2025.2548356 (2025).

